# The integrated single-cell analysis developed an immunogenic cell death signature to predict lung adenocarcinoma prognosis and immunotherapy

**DOI:** 10.18632/aging.205077

**Published:** 2023-10-04

**Authors:** Pengpeng Zhang, Haotian Zhang, Junjie Tang, Qianhe Ren, Jieying Zhang, Hao Chi, Jingwen Xiong, Xiangjin Gong, Wei Wang, Haoran Lin, Jun Li, Chenjun Huang

**Affiliations:** 1 Department of Thoracic Surgery, The First Affiliated Hospital of Nanjing Medical University, Nanjing, China; 2 Department of Lung Cancer Surgery, Tianjin Medical University Cancer Institute and Hospital, Tianjin, China; 3 First Teaching Hospital of Tianjin University of Traditional Chinese Medicine, Tianjin, China; 4 Clinical Medical College, Southwest Medical University, Luzhou, China; 5 Department of Sports Rehabilitation, Southwest Medical University, Luzhou, China

**Keywords:** lung adenocarcinoma, immunogenic cell death, signature, prognosis, immunotherapy

## Abstract

Background: Research on immunogenic cell death (ICD) in lung adenocarcinoma (LUAD) has been relatively limited. This study aims to create ICD-related signatures for accurate survival prognosis prediction in LUAD patients, addressing the challenge of lacking reliable early prognostic indicators for this type of cancer.

Methods: Using single-cell RNA sequencing (scRNA-seq) analysis, ICD activity in cells was calculated by AUCell algorithm, divided into high- and low-ICD groups according to median values, and key ICD regulatory genes were identified through differential analysis, and these genes were integrated into TCGA data to construct prognostic signatures using LASSO and COX regression analysis, and multi-dimensional analysis of ICD-related signatures in terms of prognosis, immunotherapy, tumor microenvironment (TME), and mutational landscape.

Results: The constructed signature reveals a pronounced disparity in prognosis between the high- and low-risk groups of LUAD patients. The statistical discrepancies in survival times among LUAD patients from both the TCGA and GEO databases further corroborate this observation. Additionally, heightened levels of immune cell infiltration expression are evidenced in the low-risk group, suggesting a potential benefit from immunotherapeutic interventions for these patients. The expression levels of pivotal risk-associated genes in tissue samples were assessed utilizing qRT-PCR, thereby unveiling PITX3 as a plausible therapeutic target in the context of LUAD.

Conclusions: Our constructed ICD-related signatures provide help in predicting the prognosis and immunotherapy of LUAD patients, and to some extent guide the clinical treatment of LUAD patients.

## INTRODUCTION

Lung carcinoma (LC) demonstrates a substantial global incidence and remains the predominant contributor to cancer-associated mortalities [1]. From a pathological perspective, LC can be broadly classified into two categories: small cell lung carcinoma (SCLC) and non-small cell lung carcinoma (NSCLC), with NSCLC being the more prevalent type [2]. Within NSCLC, adenocarcinoma (ADC) stands out as the predominant histological subtype, accounting for approximately 40% of all instances of LC [3]. Over the past decade, significant attention has been directed towards early surgical intervention for LC, leading to improved survival prospects for individuals diagnosed at an early disease stage [4]. However, despite the advancements in understanding LC, the aggressive nature of lung adenocarcinoma (LUAD) and its associated risks continue to adversely impact patient survival and prognostic outlooks. In recent years, there have been notable strides in utilizing targeted therapies based on biomarkers, resulting in certain improvements in the survival outcomes of LC patients. Nevertheless, the broader adoption of such treatment modalities requires further comprehensive investigation [5]. As a result, delving into additional insights regarding LUAD and the identification of emerging factors linked to patient survival prognosis assume crucial significance in enhancing patient outcomes.

The utilization of single-cell RNA sequencing (scRNA-seq) technology facilitates the examination of gene expression patterns within individual cells and the elucidation of intercellular signaling networks [6–12]. This technological innovation enables the direct quantification of the transcriptional output of neoplastic cells, thereby reflecting the integration of genetic, epigenetic, and environmental cues that modulate the behavior, adaptability, and response of cancer cells to therapeutic interventions [13–19]. The integration of clinicopathological data with scRNA-seq information derived from tumor samples holds the potential to unveil innovative diagnostic and prognostic biomarkers, as well as cell types that may prove therapeutically relevant [20–22].

Under normal circumstances, millions of cells within the bodies of healthy adult individuals undergo programmed cell death mechanisms without inciting localized or systemic inflammation [23]. However, when certain cells exhibit adequate antigenicity, such as infected or malignant cells, their demise can engender an adaptive immune response mediated by cytotoxic T lymphocytes, thereby instigating immune memory. Immunogenic cell death (ICD) assumes a pivotal role in immune surveillance; nevertheless, both pathogens and cancer cells have developed strategies to elude ICD recognition [24]. ICD is typified by the exposure and release of a multitude of damage-associated molecular patterns (DAMPs), which significantly aid dying cancer cells by fostering the recruitment and activation of antigen-presenting cells [25]. Therapeutically induced ICD can stimulate anti-cancer immune responses, thereby enhancing the therapeutic efficacy of conventional chemotherapy and radiotherapy.

This drives the necessity to investigate signatures associated with ICD in LUAD. Unraveling these signatures holds the promise of prognostic insights and informs the potential for immunotherapeutic interventions. The identification of such signatures enables the prediction of patient prognosis and facilitates the identification of individuals who might benefit from immune-based therapies. Consequently, exploring the interplay between ICD, prognosis, and immunotherapy stands to revolutionize our approach to managing LUAD and improving patient outcomes.

## MATERIALS AND METHODS

### Data search

The scRNA-seq dataset with the accession code GSE150938 was procured from the Gene Expression Omnibus (GEO) repository, encompassing a cohort of 12 samples originating from patients diagnosed with LUAD. Additionally, microarray sequencing data and clinical information were amassed from various datasets, including GSE13213 (Samples number=119), GSE26939 (Samples number=115), GSE29016 (Samples number=39), GSE30219 (Samples number=86), and GSE31210 (Samples number=227). Immunogenic cell death-related genes are summarized in the Supplementary Table 1. To ensure harmonization across different datasets and mitigate potential batch effects, a transformation to transcripts per million (TPM) format was performed on the expression data, followed by the application of the “combat” function from the “sva” package [26]. Moreover, bulk RNA-seq data, mutation profiles, and comprehensive clinical particulars of patients afflicted with lung adenocarcinoma were sourced from the TCGA database. A uniform log2 transformation was systematically applied to all datasets before embarking on the subsequent analytical procedures.

### scRNA-seq data processing

The scRNA-seq dataset underwent processing using the “Seurat” R program [27, 28]. Cells with a count below 3 and a features count below 200 were excluded. Subsequently, cells with an nFeature RNA count below 200 and mitochondrial count exceeding 10% of the sequencing count were eliminated, resulting in a total of 45,986 cells retained for further analysis. The dataset was normalized and scaled using Seurat's NormalizeData and ScaleData functions, respectively, while mitigating batch effect through CCA. The “FindVariableFeatures” algorithm was employed to identify the top 2000 variable genes. Employing Seurat’s Uniform Manifold Approximation and Projection (UMAP) technique in conjunction with the “FindClusters” function, clustering analysis was carried out with a specified resolution of 0.8. Identification of marker genes was executed utilizing Seurat’s “FindAllMarkers” function, where selection of cluster-specific markers hinged on a log2 fold change (log2 FC) threshold set at 0.25. Commonly established cell markers were harnessed for the purpose of cell classification. For quantifying gene set activity within individual cells, the “AUCell” R package was employed, computing gene expression rankings grounded on the area under the curve (AUC) value derived from model genes [29]. Cells exhibiting heightened expression levels for the targeted gene set were associated with correspondingly elevated AUC values. Determination of the threshold for discerning cells featuring active gene sets was accomplished through the utilization of the “AUCell exploreThresholds” function. Furthermore, the UMAP embedding of each cell’s AUC score was visually represented using the “ggplot2” R package, facilitating the identification of clusters characterized by active gene set profiles.

### Signature construction

The FindAllMarkers function was used to identify differential genes between high-ICD-active and low-ICD-active cell populations. These genes were subsequently integrated into transcriptome sequencing to construct a model. A total of 68 genes associated with prognosis were discerned via univariate regression analysis. To further refine the gene selection for model construction, lasso regression and multifactorial analysis were implemented [30, 31]. Risk scores were assigned to each LUAD patient utilizing the coefficients derived from the multivariate analysis. Based on the median risk score, patients from the TCGA-LUAD dataset were classified into high- and low-risk groups. Prognostic implications were evaluated by generating survival curves employing the Kaplan-Meier technique, and statistical significance was determined through log-rank tests. The evaluation of the model’s predictive efficacy was conducted through the utilization of receiver operating characteristic (ROC) curves, where a threshold AUC value exceeding 0.65 denoted exceptional performance. The validation of the predictive potential of the identified signature encompassed a comprehensive analysis across five distinct datasets from the GEO repository, involving survival analysis and AUC assessments. Furthermore, to provide a visual depiction of the patient distribution across varying risk strata, both principal component analysis (PCA) and t-distributed stochastic neighbor embedding (t-SNE) techniques were employed. The application of analogous methodologies was extended to the validation cohorts, yielding analogous insights into the distribution patterns of patients within those contexts.

### The establishment of the nomogram and evaluation

By integrating the risk score with clinical features, a more precise and refined nomogram was formulated using the ‘rms’ R package [32], resulting in the augmentation of prognostic predictive capability. The effectiveness of the nomogram was evaluated through the calculation of c-index and analysis of ROC curves. Stratified evaluations were undertaken, segregating analyses according to factors such as age, pathological T status, N status, and clinical stage. This approach served to assess the predictive relevance of both the risk score derived from the signature and the inherent clinical characteristics.

### Enrichment pathway exploration

For the GSVA analysis, integration involved the utilization of the “h.all.v7.5.1.symbols.gmt” gene sets sourced from MSigDB, accessible at the link (https://www.gseamsigdb.org/gsea/msigdb/index.jsp) [33]. Following this, the examination of gene set activities within each sample was carried out employing the GSEABase software package. Subsequently, GSEA methodology was implemented to uncover the signaling pathways and biological processes that exhibited significant enrichment within the high- and low-risk groups [34]. To gauge the enrichment scores associated with infiltrating immune cells and immunological processes, the ssGSEA method was deployed [35]. In the context of performing Kyoto Encyclopedia of Genes and Genomes (KEGG) and Gene Ontology (GO) enrichment analyses for the differentially expressed genes across the three distinct risk groups, the R packages “clusterProfiler” and “org.Hs.eg.db” were utilized [36]. Visualization of the outcomes derived from the enrichment analyses was effectively accomplished using the “ggplot2” and “GseaVis” R software packages.

### Mutations in different risk groups

The “maftools” R package was employed to analyze somatic mutations within the high- and low-risk cohorts of LUAD patients [37]. Utilizing data from the TCGA database, we generated mutation annotation format (MAF) files to capture the mutational landscape. The evaluation of tumor mutation burden (TMB) was conducted for each individual in the LUAD cohort. Visual representation of the mutation landscape alongside immune infiltration scores was achieved through the application of the “ComplexHeatmap” R package [38]. Categorization of TCGA-LUAD patients into four distinct groups was carried out, followed by the comparison of their survival disparities based on the median risk score and TMB levels.

### Immunotherapeutic strategies and assessment of the immune microenvironment

Patient data derived from the TIMER 2.0 database were procured for lung adenocarcinoma (LUAD), and the evaluation of immune cell infiltration was undertaken through the utilization of seven distinct methodologies within the TCGA dataset. Heatmaps were harnessed as a visual tool to illustrate variations in immune cell infiltration across diverse risk groups. Disparities and correlations among immune checkpoint genes specific to the high- and low-risk cohorts were systematically explored, employing boxplots and scatter plots. The quantification of enrichment scores encompassing a diverse spectrum of 29 immune signatures was accomplished through the implementation of the ssGSEA methodology. In parallel, the assessment of immunological scores, Stromal Scores, and ESTIMATE Scores for the LUAD patient cohort was executed via the utilization of the “estimate” R package [39]. Leveraging the potential to predict the responsiveness to immunotherapeutic interventions, the Cancer Immunome Atlas (TCIA) database was harnessed, serving as a repository to access the Immunophenoscores (IPS) data specific to the TCGA-LUAD population [40]. In the course of this comprehensive exploration, a rigorous juxtaposition of IPS profiles was meticulously conducted, enabling a discerning comparison between the high-risk and low-risk subgroups. This analytical endeavor elucidates valuable insights into the nuanced immunotherapeutic propensities residing within these distinct risk categories.

### qRT-PCR assay

The process of extracting total RNA from LUAD tissues was successfully executed through the utilization of TRIzol reagent (Thermo Fisher Scientific, Waltham, MA, USA). In strict adherence to the stipulated protocols provided by the manufacturer, the cDNA synthesis step was performed, facilitated by the RevertAid™ First Strand cDNA Synthesis Kit (Thermo Fisher Scientific). Subsequently, the application of a qRT-PCR assay transpired, employing the StepOne Real-Time PCR system (Thermo Fisher Scientific) in conjunction with a SYBR Green PCR kit (Takara Bio, Otsu, Japan). In the pursuit of quantifying the levels of relative gene expression, the 2^-ΔΔCT^ method was aptly engaged. This systematic procedure underscores the meticulous attention to methodological precision, ensuring the robustness and reliability of the ensuing molecular analyses.

### Statistical analysis

R version 4.1.3 was the platform employed for all statistical analyses and data processing endeavors. Survival analysis encompassed the utilization of Kaplan-Meier curves, with the determination of statistical significance achieved through application of the log-rank test. The generation of survival curves was facilitated by the utilization of the “survminer” R package [41]. Furthermore, the creation of heatmaps was executed using the “Pheatmap” R package. For variables exhibiting a normal distribution, quantitative disparities were evaluated using either a two-tailed t-test or a one-way analysis of variance. Non-normally distributed data was subjected to analysis through either the Wilcoxon test or the Kruskal-Wallis test. The entire spectrum of statistical analyses was undertaken within the R environment, with statistical significance deemed present at a threshold of P < 0.05.

### Availability of supporting data

The datasets analyzed in the current study are available in the TCGA repository (http://cancergenome.nih.gov/), and GEO (https://www.ncbi.nlm.nih.gov/geo/).

### Consent for publication

All authors consent to the publication of this study.

## RESULTS

### Cells clustering results and AUC value calculation

**Figure 1:**
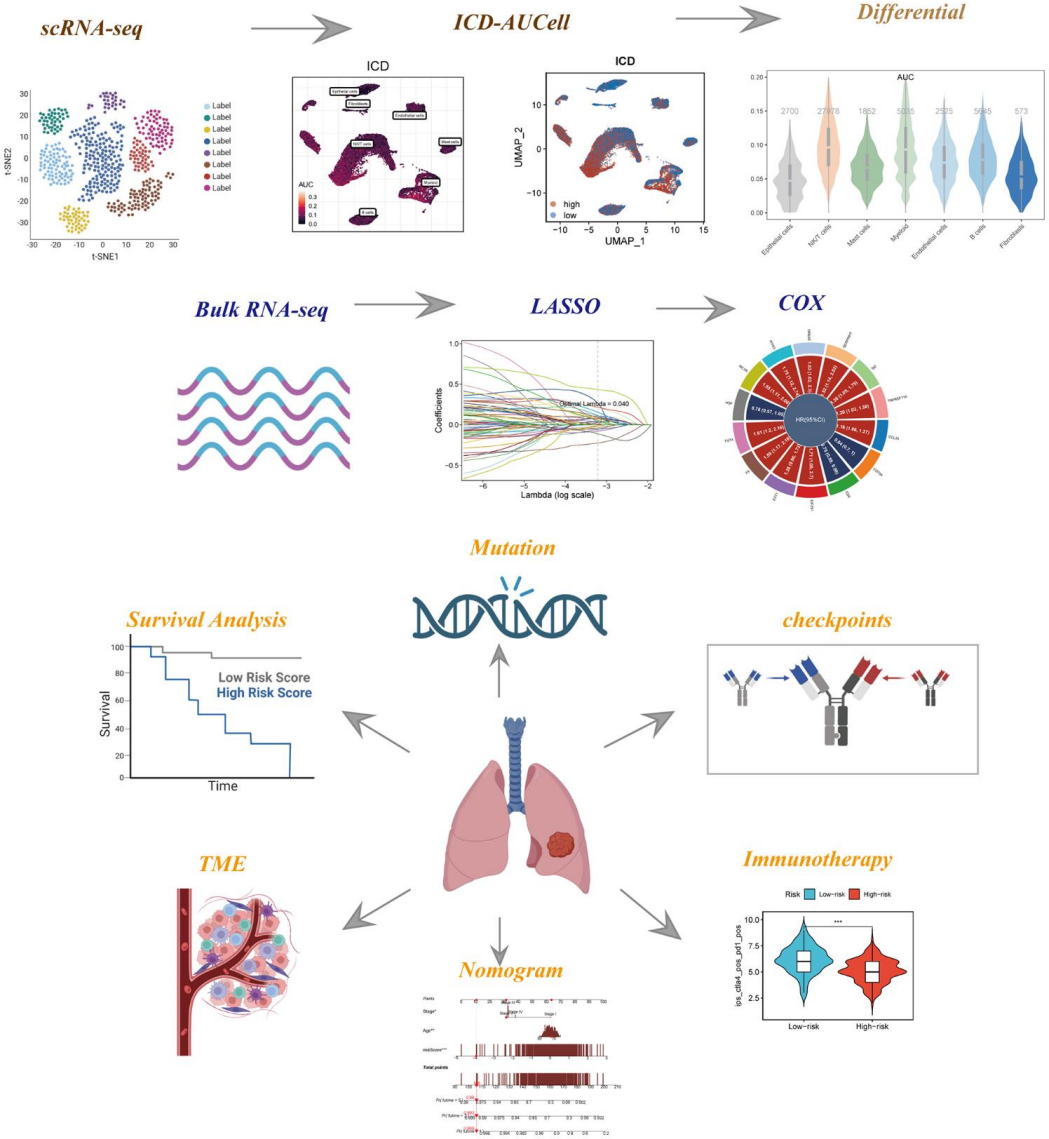
**The flowchart of this investigation.** To integrate multiple datasets to explore the role of ICD in the TME of LUAD, and to construct a signature to predict the prognosis and immunotherapy of LUAD patients.

**Figure 2:**
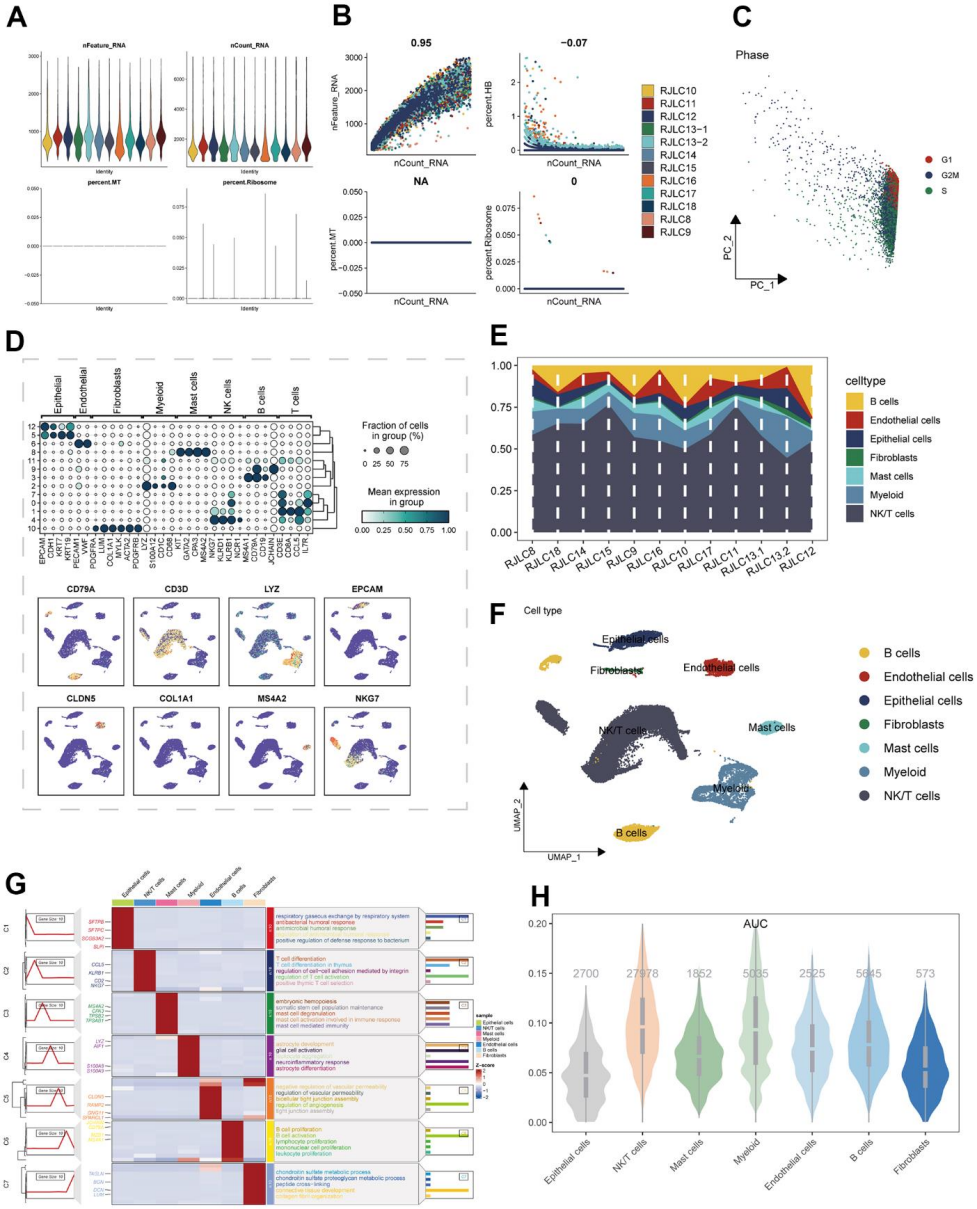
The single-cell analysis process was executed as follows: (**A**) The distribution of gene expression levels, sequencing depth, the percentage of red blood cell genes, the percentage of mitochondrial genes, and the percentage of ribosome genes was assessed across the 12 samples. (**B**) Correlations between sequencing depth and gene expression levels, as well as the percentages of mitochondrial genes, red blood cell genes, and ribosome genes, were examined. (**C**) The distribution of observation samples slated for clustering based on cycle-related marker scores was analyzed. (**D**) The expression of marker genes within diverse subpopulations of classical cell types was depicted. (**E**) The distribution of distinct cell populations across the 12 LUAD samples was illustrated. (**F**) An UMAP plot displayed the comprehensive composition of cell types. (**G**) Differential expression markers between various enriched cell types, aligned with pathway profiles, were identified. (**H**) Divergent ICD activity within distinct cell groups was ascertained.

The study’s schematic representation was visually outlined in Figure 1. Depiction of gene expression level distributions, sequencing depth characteristics, proportions of red blood cell genes, mitochondrial gene proportions, and ribosome gene proportions across the 12 samples was presented in Figure 2A. The visualization of correlations between sequencing depth and gene expression levels, along with the proportions of mitochondrial genes, red blood cell genes, and ribosome genes, was rendered in Figure 2B. Following meticulous data processing and rigorous filtration, gene expression profiles derived from a collective total of 45,986 cells spanning across 12 LUAD samples were obtained for subsequent comprehensive analysis. The consistent distribution of cells within each individual sample indicated a notable absence of pronounced batch effects, a beneficial facet for forthcoming investigations (Supplementary Figure 1A). The PCA reduction plot, depicted in Figure 2C, showed that there were no apparent distinctions observed among the various cell cycles. Subsequently, employing UMAP as the dimensionality reduction method, all cells were classified into 13 clusters (Supplementary Figure 1B). The bubble and UMAP plots in Figure 2D displayed the typical marker genes for different cell types and their corresponding clusters. Figure 2E presented the proportion of each cell type within each of the 12 samples, while Figure 2F showed the distribution of each cell type. Figure 2G illustrated the differential genes between the different cell types, with the top 4 markers randomly labeled on the left side, and the right side utilizing GO enrichment analysis to demonstrate the pathways in which the marker genes were primarily enriched. Finally, the ICD activity was calculated as a gene set for each cell, and Figure 2H displayed the variation in ICD activity among different cell types, indicating relatively higher ICD activity in NK/T cells and Myeloid cell types.

### Identifying the genes most relevant to ICD

**Figure 3:**
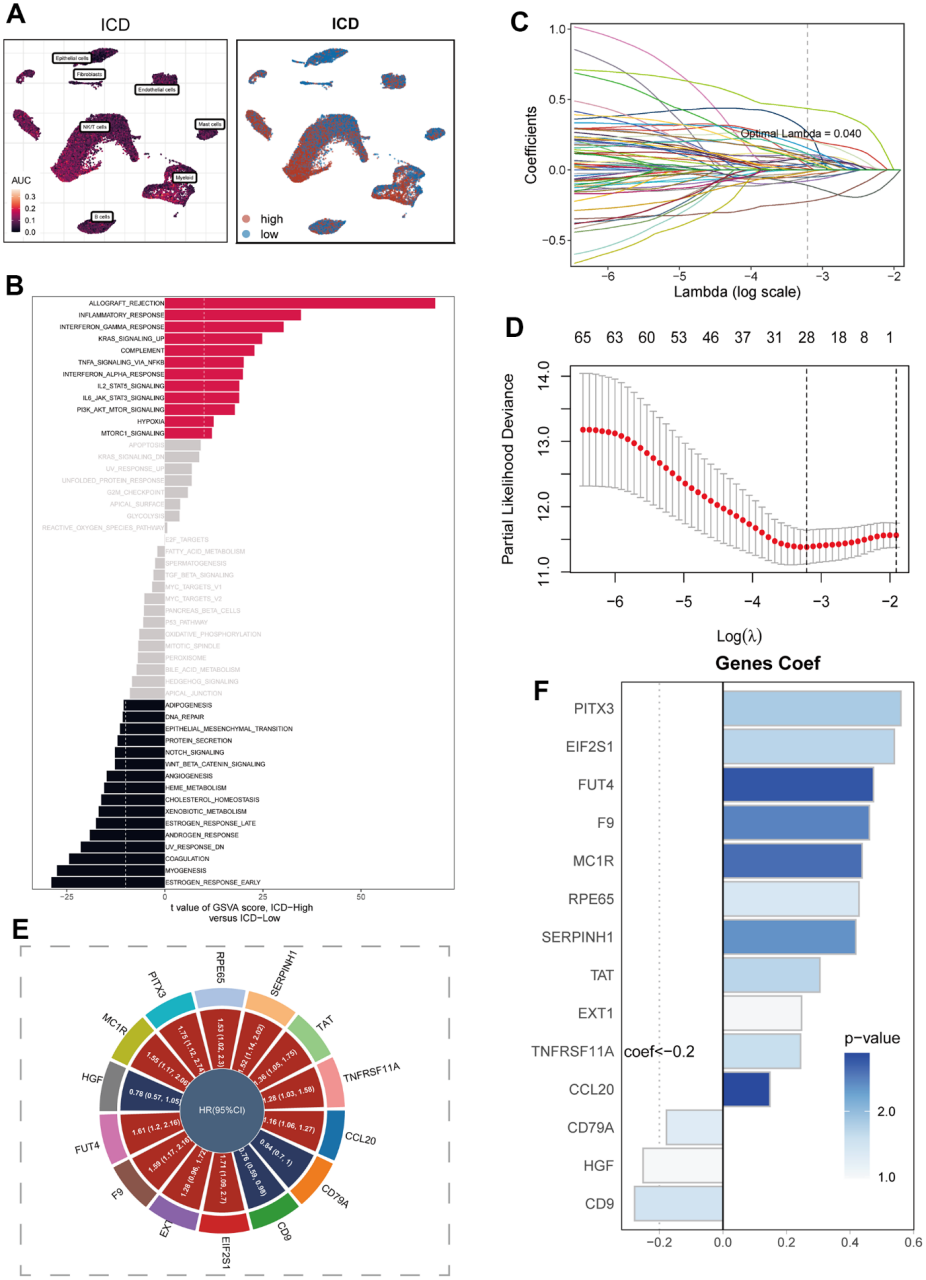
**Construction of the signature.** Model construction. (**A**) Distribution of ICD activity in the cell population. (**B**) GSVA enrichment analysis between high and low ICD groupings. (**C**, **D**) Variable screening and model construction for differential genes between high and low ICD subgroups using lasso regression. (**E**) HR values of model genes. (**F**) Coefficients of model genes.

In Figure 3A, the visualization of ICD activity in cells was presented using the AUCell algorithm. In order to offer a more visual portrayal of the ICD scores in cells, the cells were dichotomized based on the median value of these scores. Through the execution of GSVA enrichment analysis, the observation emerged that cells categorized within the high ICD group manifested noteworthy enrichment in pathways related to Allograft Rejection, Inflammatory Response, and Interferon Gamma Response (Figure 3B). In contrast, the Estrogen Response Early pathway demonstrated marked enrichment in the low ICD group. Moving forward, the marker genes that exhibited differential expression in the high and low ICD groups, as acquired from Bulk RNA-seq data, were harnessed to formulate prognostic models via COX and Lasso regression analyses. The process of identifying pivotal prognostic variables was depicted in Figure 3C, 3D. The subsequent Figure 3E depicted the hazard ratio (HR) values corresponding to each variable integrated into the model, while Figure 3F showcased the coefficients attributed to specific variables within the model.

### Evaluation of the model

**Figure 4:**
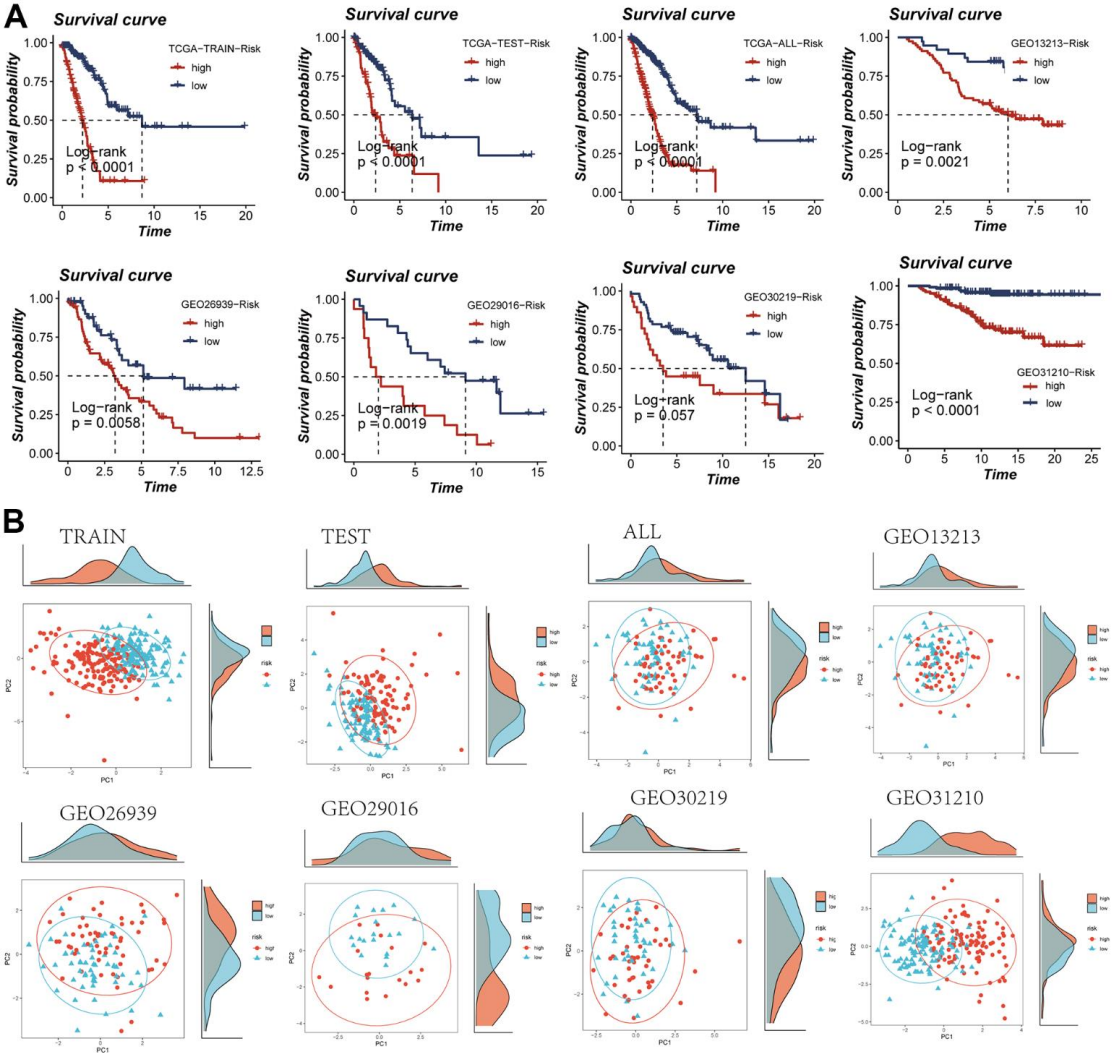
**Assessment of risk models.** (**A**) Kaplan-Meier survival analysis of signatures in the TCGA and five GEO datasets. (**B**) Observation of the distribution of samples in high- and low-risk groups using PCA analysis.

The computation of individual patient risk scores entailed the multiplication of model gene expression values by their respective coefficients. Subsequent to this, patient stratification into high and low-risk categories was conducted based on the median risk score value. It is noteworthy that both the TCGA dataset and the five GEO cohorts consistently exhibited inferior prognosis outcomes within the high-risk group, thereby substantiating the precision and robustness of the developed model (Figure 4A). The distribution of samples was visually presented through a PCA plot, elucidated in Figure 4B. Furthermore, the spatial arrangement of samples within the high- and low-risk groups was depicted in the t-SNE plot, accessible in Supplementary Figure 2.

### The construction of a nomogram

**Figure 5:**
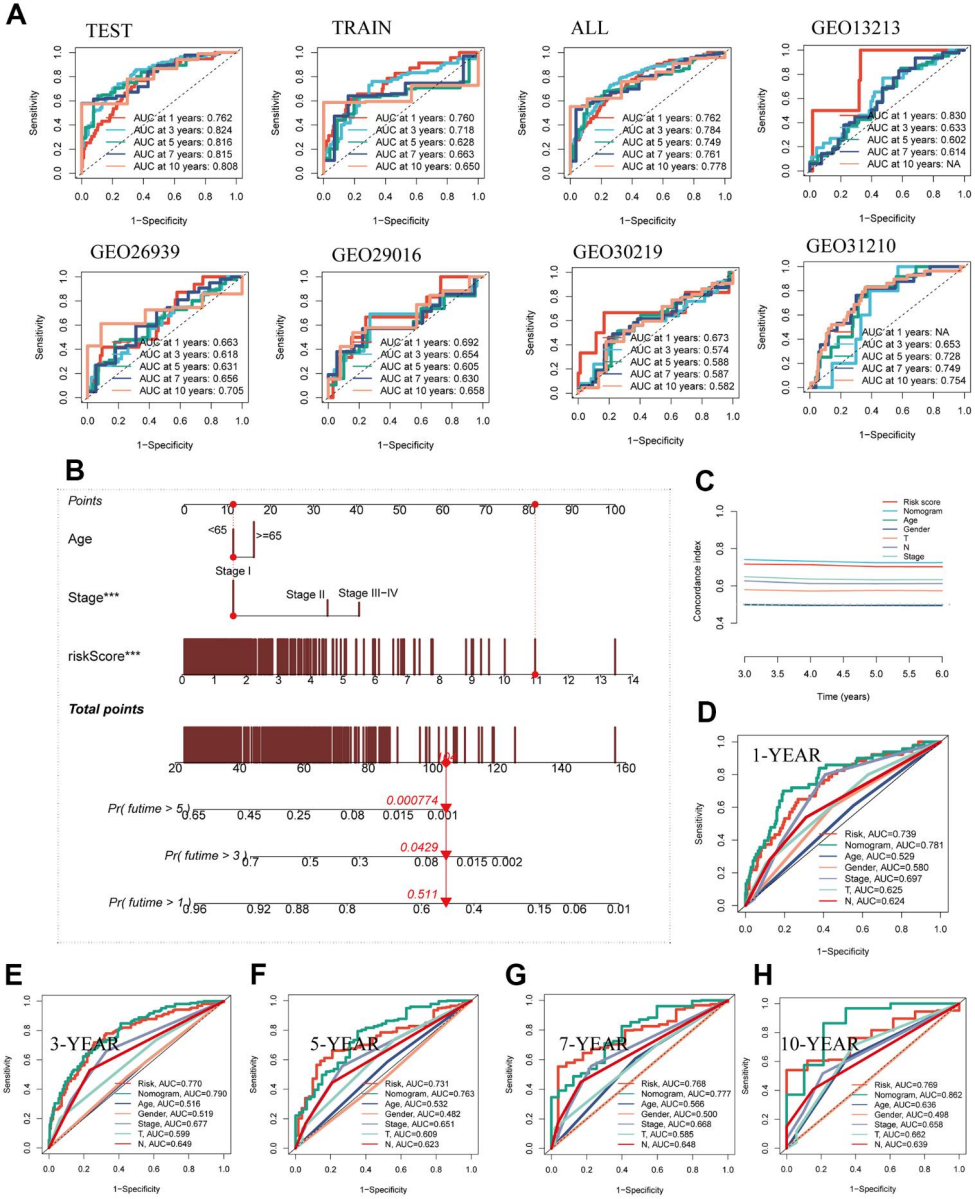
**Building a more accurate nomogram.** (**A**) The ROC curve was used to evaluate the performance of the model in the TCGA and five GEO datasets. (**B**) Nomogram was constructed by combining clinical features with risk groups. (**C**) C-index curves were utilized to evaluate the predictive performance of different clinical characteristics, nomogram scores, and risk scores. (**D**–**H**) ROC curves for 1, 3, 5, 7, and 10 years showed AUC values for various clinical factors, risk scores, and nomogram scores.

The predictive performance of the model regarding prognosis was meticulously evaluated through the utilization of ROC curves across both the TCGA dataset and the five GEO datasets, thereby substantiating the robustness and consistency of our developed model within the ambit of multi-dataset validation. Remarkably, a substantial majority of the AUC values surpassed the threshold of 0.6 across both the TCGA and GEO datasets, as evidenced in Figure 5A. To comprehensively assess the risk associated with TCGA-LUAD patients, a nomogram was meticulously formulated, integrating crucial clinical features alongside risk group stratification. As depicted in Figure 5B, this nomogram visually encapsulates patient stage, age, and risk score, thereby serving as a valuable tool for enhanced precision in risk assessment and informing forthcoming treatment strategies. Inclusive of C-index curves, the analysis demonstrated the superior predictive performance of the nomogram scores when compared against alternative clinical features and risk scores, as illustrated in Figure 5C. Moreover, the nomogram’s accuracy was methodically gauged via prognostic ROC analysis, consistently showcasing markedly superior performance in comparison to other clinical features and risk scores. Notably, the AUC values for various time horizons including 1, 3, 5, 7, and 10 years were 0.781, 0.790, 0.763, 0.777, and 0.862, respectively, underscoring the substantial predictive capability of the nomogram across diverse temporal scopes (Figure 5D–5H).

### Relationship between model and clinical characteristics

**Figure 6:**
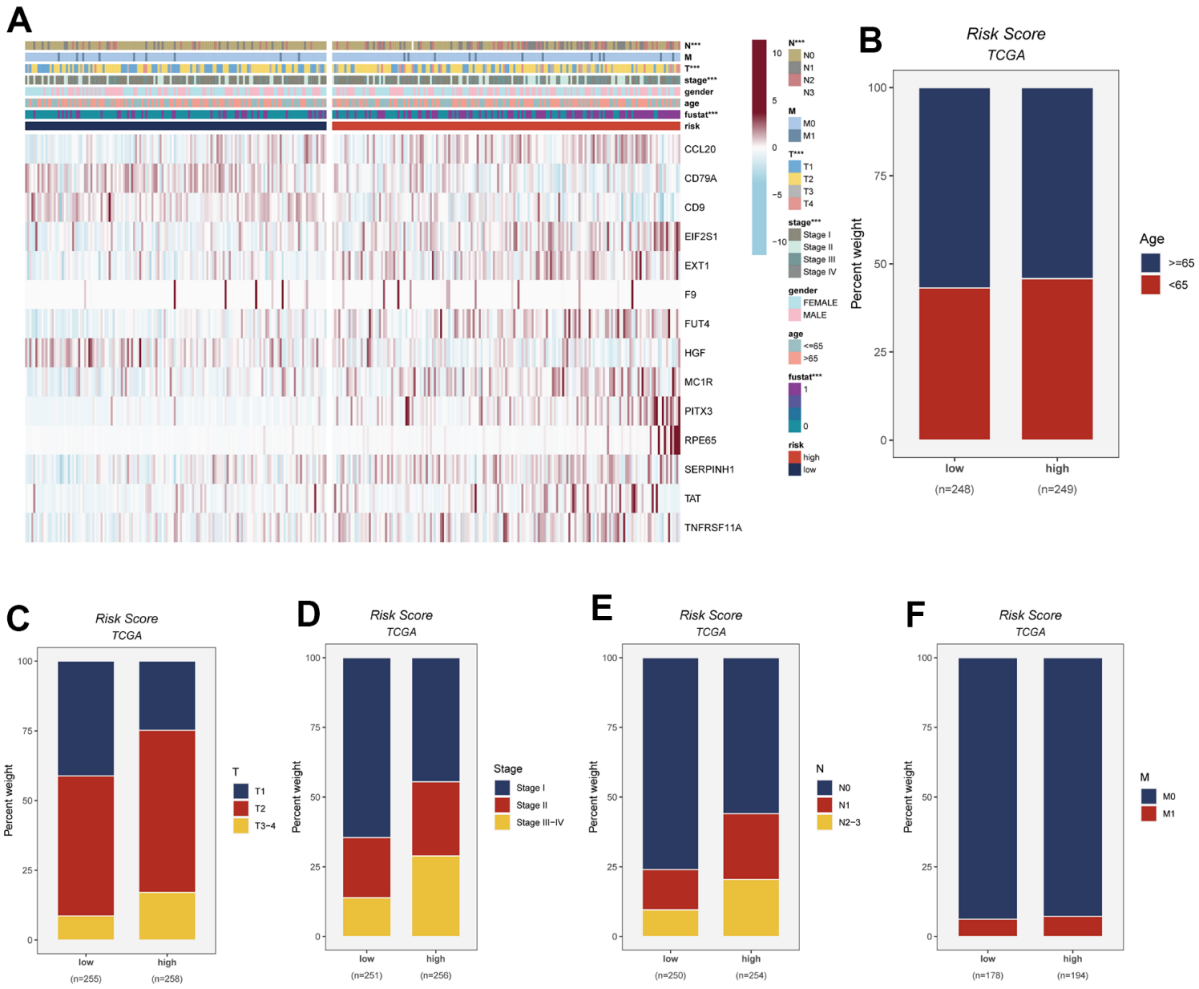
**This figure provides insights into the connection between risk scores and various clinical attributes.** The specific details are as follows: (**A**) Clinical characteristics are integrated with risk grouping to facilitate an examination of the distribution of clinical attributes and the expression patterns of model genes across distinct risk groups. (**B**–**F**) The distribution of age, T-stage, clinical stage, N-stage, and M-stage among LUAD patients in different risk groups is observed and presented as percentages.

To visually depict the distribution of clinical attributes within distinct risk subgroups, a heatmap was meticulously generated. This heatmap seamlessly amalgamates pertinent clinical information with the high- and low-risk subgroups, as vividly depicted in Figure 6A. Notably, the high-risk cohort exhibited a discernible association with more advanced T-stage, N-stage, and clinical stage statuses. These findings, showcased in Figure 6B–6F, collectively signify an unfavorable prognostic outlook for patients encompassed within the high-risk subgroup.

### Functional analysis of ICD-related signatures

**Figure 7:**
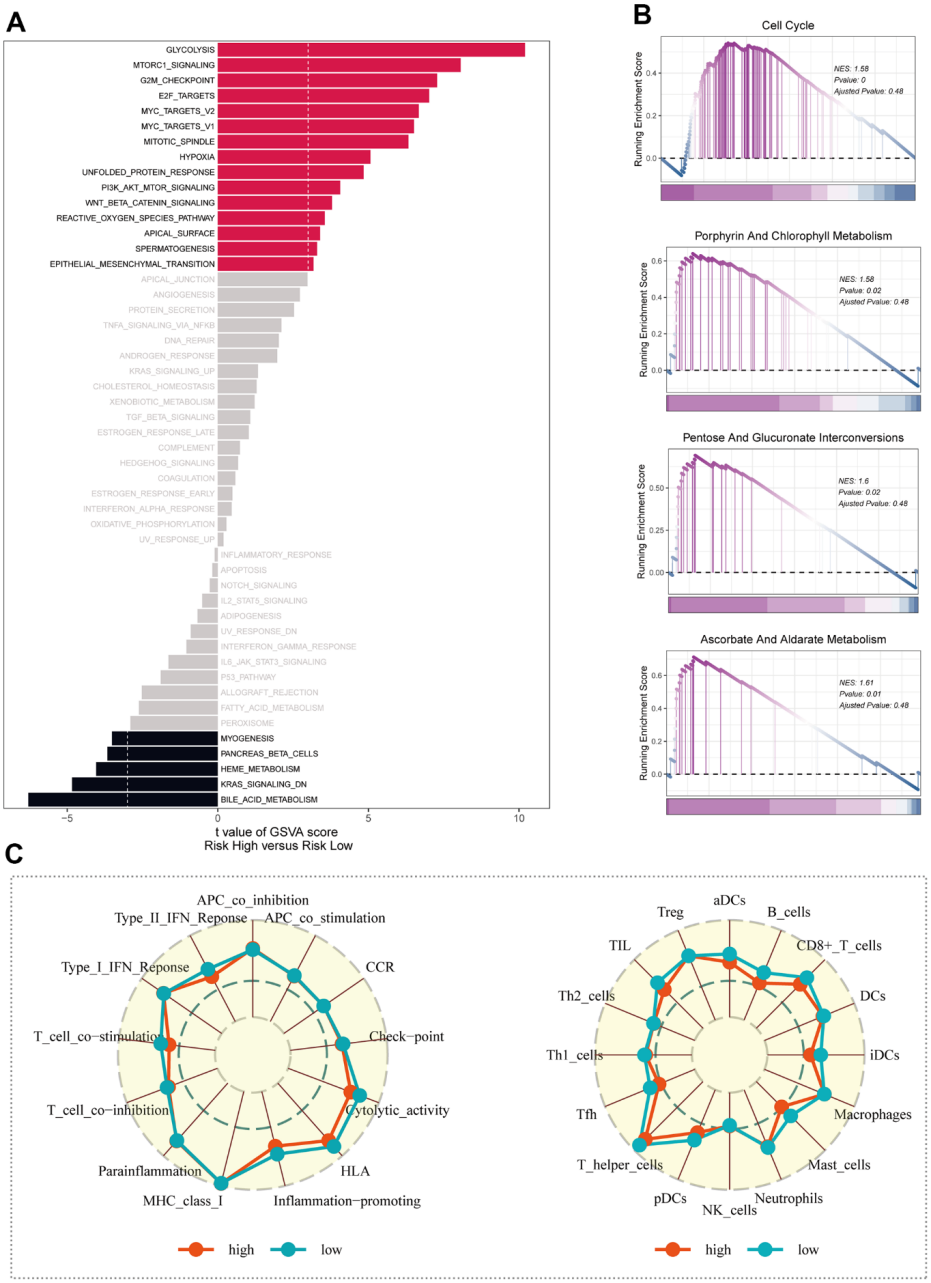
**Enrichment analysis.** (**A**) Exploring differences in GSVA enrichment analysis across risk groups. (**B**) Enrichment analysis of KEGG for differential genes in high and low risk groups. (**C**) Study of immune cell infiltration and immune-related pathway enrichment in high- and low-risk groups using ssGSEA.

The GSVA results unveiled that considerable enrichment in pathways such as glycolysis, mTORC1 signaling, and the G2M checkpoint was evident among patients belonging to the high-risk group (Figure 7A). The KEGG enrichment analysis map visually showcased significant enrichment in pathways like porphyrin and chlorophyll metabolism, alongside pentose and glucuronate interconversions (Figure 7B). Through ssGSEA analysis of the corresponding data, it was observed that the low-risk group exhibited a greater abundance of infiltrating immune cells, including B-cells and aDCs (Figure 7C).

### Assessment of immune microenvironment

**Figure 8:**
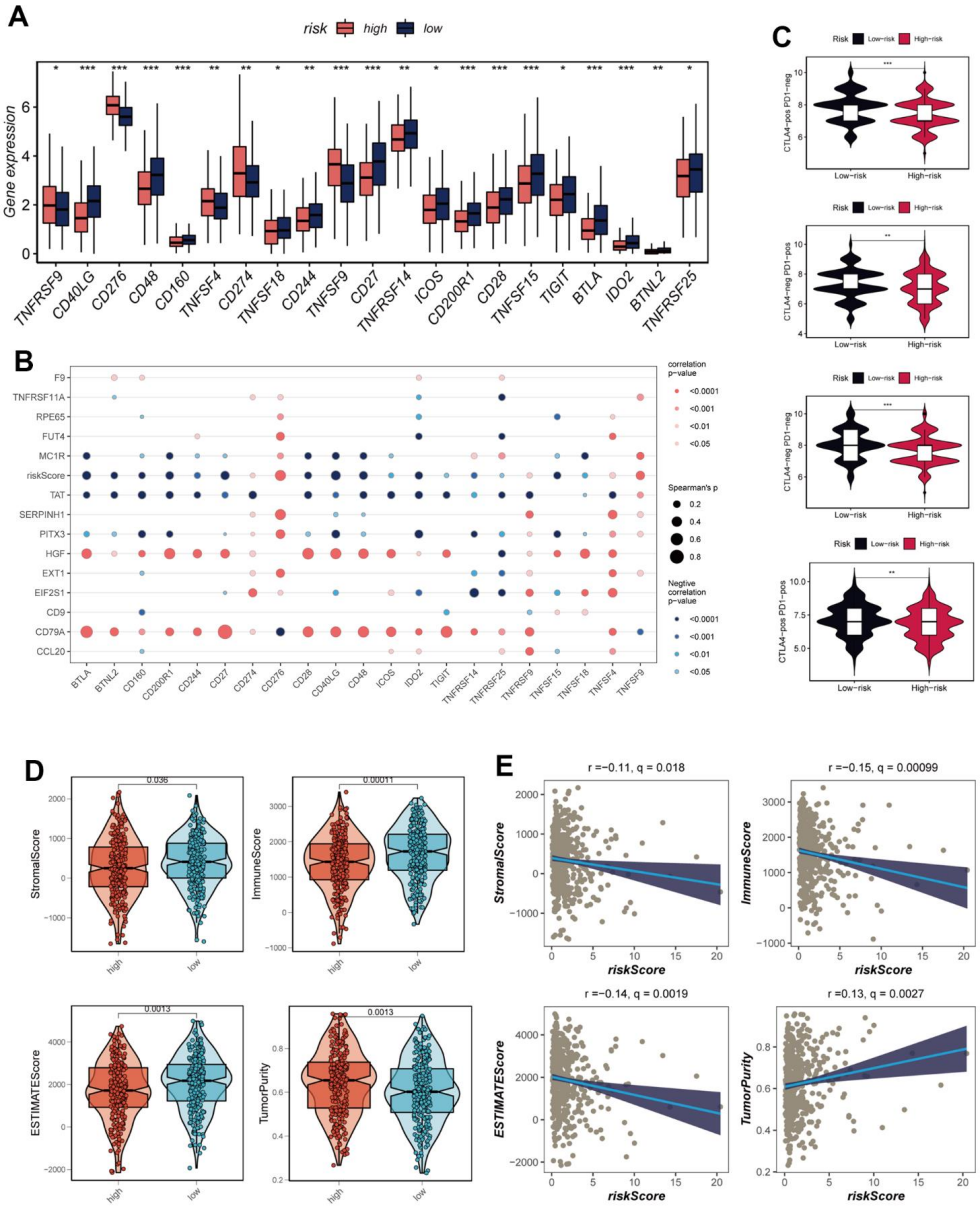
**Analysis of immune cell content and immune checkpoint.** (**A**) A box plot showed that differences in immune checkpoint gene expression between high- and low-risk groups. (**B**) Correlation between model genes and immune checkpoint. (**C**) The low-risk group has significantly greater IPS, IPS-CTLA4-neg-PD-1-neg, IPS-CTLA4-pos-PD-1-neg, IPS-CTLA4-neg-PD-1-pos, and IPS-CTLA4-pos-PD-1-pos. (**D**) The violin plot demonstrated the difference in Stromal Score, Immune Score, ESTIMATE Score, and tumor purity calculated using the ESTIMATE algorithm between the two risk subgroups. (**E**) The correlations in Stromal Score, Immune Score, ESTIMATE Score, and tumor purity were calculated using the ESTIMATE algorithm between the two risk subgroups. Note: *P < 0.05, **P < 0.01, ***P < 0.001.

The utilization of immune checkpoint blockade has been extensively integrated into the treatment paradigm for advanced LUAD patients. An in-depth analysis was conducted to explore the intricate relationship between risk scores and established immune checkpoint genes (ICGs) within the TCGA-LUAD cohort. Remarkably, discernible trends emerged as the low-risk group exhibited elevated expression levels across a multitude of immune checkpoint genes including CD48, CD40LG, and CD27, as visually represented in Figure 8A. Interactions between model genes, risk scores, and ICGs were thoroughly examined, the outcomes of which were encapsulated within bubble plots for visualization purposes (Figure 8B). The shade of blue denoted negative correlation, while orange hues represented positive correlation. Intriguingly, a positive correlation emerged between the expression levels of model genes and a significant proportion of immune checkpoints. In stark contrast, risk scores displayed a negative correlation with select prevalent immune checkpoint genes, notably BTLA, BTNL2, CD160, and CD244. These revelations collectively underscore the potential promise of immune checkpoint blockade therapy for individuals afflicted by LUAD. To gauge the potential therapeutic implications of immunotherapy within distinct risk strata, an assessment of immune checkpoint inhibitor response prediction scores (IPS) was executed across diverse risk categories. This endeavor aimed to pinpoint those patients who might derive enhanced benefits from immunotherapeutic interventions. The analysis deduced that tumor samples from patients in the low-risk group were more likely to exhibit favorable immune responses to PD-1/PD-L1 or CTLA4 inhibitors, or a combination thereof (Figure 8C). Substantiated by notably higher IPS scores, the low-risk group emerged as candidates with the greatest potential gains from this therapeutic approach. Exploiting data from seven immune infiltration algorithms resident within the TIMER database, an extensive evaluation was carried out to discern discrepancies in immune infiltration between the high- and low-risk groups within the TCGA-LUAD context, as revealed in Supplementary Figure 3. Furthermore, the ESTIMATE methodology was harnessed to validate immune infiltration levels across distinct risk groups. Spearman correlation analysis was engaged to elucidate the intricate interplay between risk scores and immune infiltration scores. Evidently, the low-risk cohort exhibited heightened Immune Scores (P<0.05), as depicted in Figure 8D. Significantly, a notable negative correlation emerged between risk scores and Immune Scores (R = -0.15, FDR<0.001, Figure 8E), underscoring the intrinsic association between risk scores and the extent of immune cell infiltration within the TME. This dynamic relationship might contribute to variances in disease progression and the efficacy of immunotherapy in LUAD patients.

### Association between mutation and prognosis

**Figure 9:**
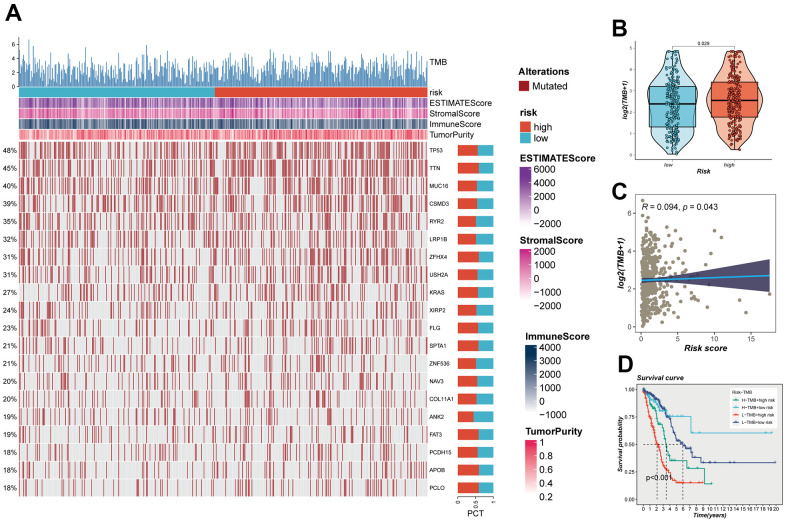
**Mutation analysis.** (**A**) Mutation landscape of the top 20 genes with mutation frequency in the high-low risk group. (**B**) TMB differences between high- and low-risk patients. (**C**) Relationship between risk score and tumor mutation burden. (**D**) Survival analysis between four different groups (High-TMB+High-Risk, High-TMB+Low-Risk, Low-TMB+High-risk, and Low-TMB+low-risk).

The impact of somatic mutations on the outcomes of cancer immunotherapy is inherently variable. The mutational landscape inherent to TCGA-LUAD was subject to meticulous examination, with the outcomes comprehensively illustrated in Figure 9A. Furthermore, an intricate and systematic juxtaposition was orchestrated to meticulously unravel the variances in TMB that might distinguish the high and low-risk groups. Evidently, this analytical endeavor unveiled a discernible augmentation in the frequency of mutations within the high-risk contingent of LUAD (Figure 9B). This assertion was corroborated through an intensive Spearman correlation analysis, which underscored a statistically significant and positive correlation between risk scores and TMB (R = 0.094, P = 0.043, Figure 9C). Utilizing a pragmatic approach predicated on the median benchmarks of TMB and risk scores, a judicious stratification of LUAD patients occurred, subsequently yielding four distinct categories: high-mutation+high-risk, high-mutation+low-risk, low-mutation+high-risk, and low-mutation+low-risk. The ensuing elucidation of outcomes distinctly delineated that individuals contending with high-mutation+low-risk LUAD exhibited a notably promising prognostic trajectory, in stark contrast to their counterparts grappling with low-mutation+high-risk LUAD, who faced a markedly less favorable prognostic landscape (Figure 9D).

### The role of PITX3

**Figure 10:**
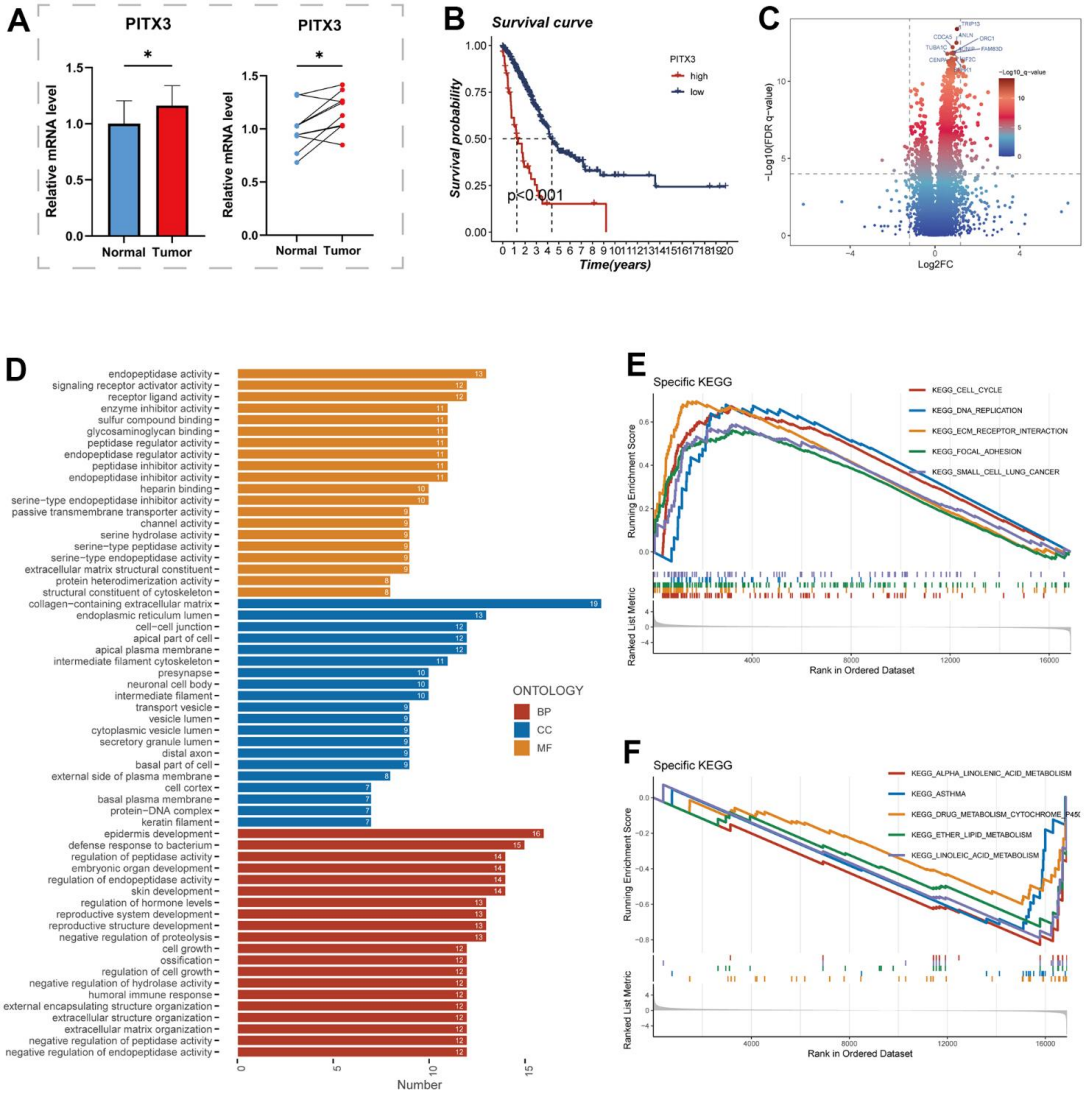
**The role of PITX3.** (**A**) Differential expression of PITX3 in normal and tumor samples of TCGA. (**B**) Survival differences in high and low PITX3-expressing samples. (**C**) Differential genes between samples with high and low expression of PITX3, results are presented in volcano plots. (**D**) Pathways of differential gene enrichment in GO. (**E**, **F**) GSEA enrichment analysis in the high- and low-PITX3 expressing groups.

For further analysis, the PITX3 gene, which exhibited the highest HR value, was selected from the model gene. Figure 10A illustrates the elevated expression levels of PITX3 in tumor samples. Additionally, the high expression of the PITX3 gene was associated with a poorer prognosis compared to the low expression group (*P*<0.001, Figure 10B). These conspicuous differences align with our bioinformatic findings, suggesting that PITX3 could serve as a promising biomarker for early detection of LUAD. Subsequently, LUAD patients were divided into high and low expression groups based on PITX3 expression, and differential analysis was performed. Figure 10C showcases the differentially expressed genes, highlighting the top 10 genes with the most significant differences. Furthermore, GO and GSEA enrichment analyses of these differentially expressed genes revealed significant enrichment in pathways related to collagen-containing extracellular matrix and cell-cell junctions in the highly expressed PITX3 group (Figure 10D, 10E).

## DISCUSSION

Despite the progress achieved in comprehending LC’s susceptibility, progression, immune regulation, and treatment options, it continues to retain its position as the primary cause of cancer-related mortality [42]. NSCLC represents the most common form within the pathological classification of LC. Specifically, LUAD is the predominant subtype of NSCLC and the most frequent primary LC [43]. Despite ongoing exploration and research in LC treatment, the lack of reliable early prognostic indicators hinders treatment efficacy [44]. Regulated cell death (RCD), a form of cell death controlled by gene-encoded molecular mechanisms, has long been recognized as an event associated with immune suppression or even tolerance [45]. Releasing a diverse array of damage-associated molecular patterns (DAMPs), the phenomenon of RCD operates to orchestrate the recruitment and activation of antigen-presenting cells, thereby offering significant potential for the development of innovative approaches to cancer therapy [25]. The overarching concept of cancer immunotherapy aims to harness the immune system's intrinsic capabilities to induce an immune response against tumors. Through the initiation of ICD, the sustained effectiveness of anticancer medications can be attained, thereby merging direct elimination of cancer cells with the stimulation of antitumor immune responses [46]. Noteworthy antecedent investigations have underscored the prognostic significance of genes implicated in ICD within patients afflicted by head and neck squamous cell carcinoma (HNSCC) [47, 48]. However, the precise role of ICD within the context of LUAD remains enigmatic. The primary goal of this study is to meticulously unravel the prognostic implications and therapeutic prospects that ICD potentially harbors within the realm of LUAD.

Unprecedented resolution is provided by single-cell analysis for the exploration of intratumoral heterogeneity, cell differentiation trajectories, and intercellular communication, offering a range of promising applications [29]. By examining cell clustering within scRNA-seq datasets, genes exclusively expressed in tumor cells were identified. This shift redirects attention away from conventional comparisons between tumors and normal tissues, as observed in previous database analyses, towards the investigation of disparities among tumor cells themselves [30]. In this investigation, pivotal regulatory genes governing ICD were pinpointed through the utilization of scRNA-seq data. Leveraging clinical data, a prognostic signature composed of 14 genes was formulated. Subsequent calculations of risk scores facilitated the categorization of LUAD patients into high-risk and low-risk groups. When comparing survival curves between the high-risk and low-risk cohorts, a favorable prognostic trend was evident for patients within the low-risk group in the TCGA cohort (*P*<0.0001). Consistent survival outcomes were also observed in the TCGA-test group, TCGA-train group, and GEO verification group (*P*<0.05). However, no statistically significant differentiation between the high-risk and low-risk cohorts was observed within the GEO30219 group (*P*=0.057). The considerable accuracy of the constructed prognostic signature in assessing the prognosis of LUAD patients within intervals of 1, 3, 5, 7, and 10 years in both the TCGA and GEO cohorts was confirmed through ROC curve analysis. Impressively, these findings closely aligned with the predictions of the nomogram.

Functional enrichment analysis unveiled significant enrichment of ICD-related signatures in the glycolysis, mTORC1-signaling, and G2M-checkpoint pathways. Altered energy metabolism, such as glycolysis, represents a distinct feature of cancer cells, serving as one of the “hallmarks of cancer.” Glycolysis metabolism provides selective advantages to cancer cells in environments with limited nutrient supply [49]. TBK1 inhibition impaired CRC cell proliferation, migration, drug resistance, and tumor growth. Overexpression of TBK1 inhibited mTORC1-signaling activation in CRC [50]. Although there is no clear evidence that mTORC1-signaling is involved in the progression of LUAD, we can speculate that mTORC1-signaling also plays an important role in LUAD. Significant prominence is accorded to the G2M-CHECKPOINT pathway in a spectrum of related malignancies, encompassing pancreatic cancer [51], colorectal cancer [52], breast cancer [53], among others. It is discernible that the intricate regulatory mechanisms governing the G2M-CHECKPOINT pathway might equally exert an influential impact on the trajectory of LUAD progression.

An all-encompassing scrutiny of the immune cell infiltration within TME can serve as a conduit for comprehending the intricate mechanisms underpinning cancer's evasive maneuvers against immune responses, thereby opening avenues for the formulation of innovative therapeutic strategies [54]. The meticulous investigation of immune cell infiltration disparities between the high-risk and low-risk cohorts unveiled a conspicuous augmentation of immune cell abundance within the low-risk group in contrast to their high-risk counterparts. It is noteworthy that prior investigations have underscored the interrelation between immune checkpoint gene expression levels and the efficacy of immunotherapeutic interventions [55]. Delving into the variations of immune checkpoint genes between the high-risk and low-risk groups proposed the potential for the low-risk group to glean added therapeutic advantage from targeting supplementary immune checkpoints. Within the realm of cancer treatment, therapeutic modalities geared towards the TME have risen to prominence, recognized for their promise in leveraging the pivotal role that the TME holds in steering tumor progression and influencing responses to standard therapeutic regimens [56–59]. Notably, assessment of TME scoring unveiled a notable elevation in immune scores within the TME of the low-risk cohort, distinct from their high-risk counterparts, with a statistically significant divergence. This compelling observation lends credence to the notion that patients harboring low-risk profiles might exhibit heightened responsiveness to immunotherapy. Adding to these insights, a systematic analysis appraising the comparative immunotherapy advantages between the high-risk and low-risk groups corroborated the prospective amplified therapeutic gains for individuals encompassed within the low-risk classification.

Although PITX3’s role in brain development is well established, its involvement in tumorigenesis remains largely unknown. Reports have indicated high methylation levels of PITX3 in breast cancer [60]. PITX3 promoter methylation also impacts the recurrence-free survival of prostate cancer patients [61]. However, the precise role of PITX3 in LUAD progression has yet to be elucidated. Our study revealed that PITX3 exhibited the highest HR among all prognostic signature genes. PITX3 may contribute to LUAD progression. The survival analysis yielded compelling evidence linking heightened PITX3 expression with unfavorable prognostic outcomes among LUAD patients, thus underlining its potential as a viable therapeutic target. Of noteworthy significance, the ECM-receptor interaction pathway emerges as a pivotal participant in a multitude of tumor-associated processes, encompassing shedding, adhesion, degradation, motility, and proliferation [62]. This pathway's pivotal role in tumor invasion and metastasis has been decisively affirmed within the context of gastric cancer [63]. Furthermore, comprehensive investigations have unequivocally demonstrated that the intricate interplay between the extracellular matrix (ECM) and cellular receptors constitutes a fundamental conduit underpinning the progression and metastasis of colorectal cancer [64]. Within the high-risk subgroup, PITX3 manifested conspicuous enrichment within the ECM-receptor interaction pathway. This observation notably intimates that PITX3's influence might extend to interfering with the trajectory of LUAD progression by active involvement within the ECM-receptor interaction pathway, potentially engendering adverse prognostic implications for patients grappling with LUAD.

Despite its limitations, valuable insights are offered by this study into the expression of prognostic genes associated with LUAD, thereby laying the groundwork for subsequent investigations into their underlying molecular mechanisms.

In summary, the ICD-related signatures formulated within this study facilitate the prognostic prediction of LUAD patients grounded in the expression of pertinent genes. Furthermore, this work paves the way for potential applications of immunotherapy in the realm of LUAD treatment. It is important to note, however, that further experimental endeavors are warranted to substantiate and validate these findings.

## Supplementary Material

Supplementary Figures

Supplementary Table 1
