# Supplementary material for: The integrated single-cell analysis developed an immunogenic cell death signature to predict lung adenocarcinoma prognosis and immunotherapy
Source: Aging (Albany NY). 2023 Oct 4;15(19):10305–29. doi: 10.18632/aging.205077 (PMC10599752; doi:10.18632/aging.205077)
Supplement: Supplementary Figures [file aging-15-205077-s001.pdf]

SUPPLEMENTARY FIGURES

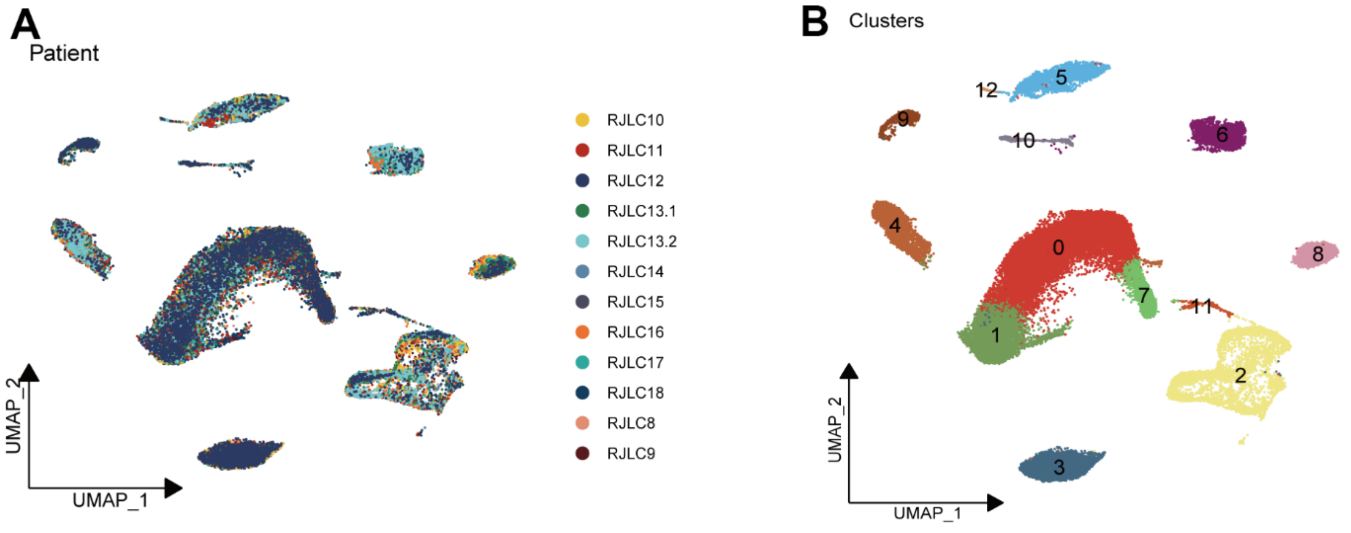

Supplementary Figure 1. Single-cell dimensionality reduction clustering. (**A**) A UMAP plot showing the cell distribution characteristics in 12 samples. (**B**) A UMAP plot showing all cells were clustered into 13 clusters.

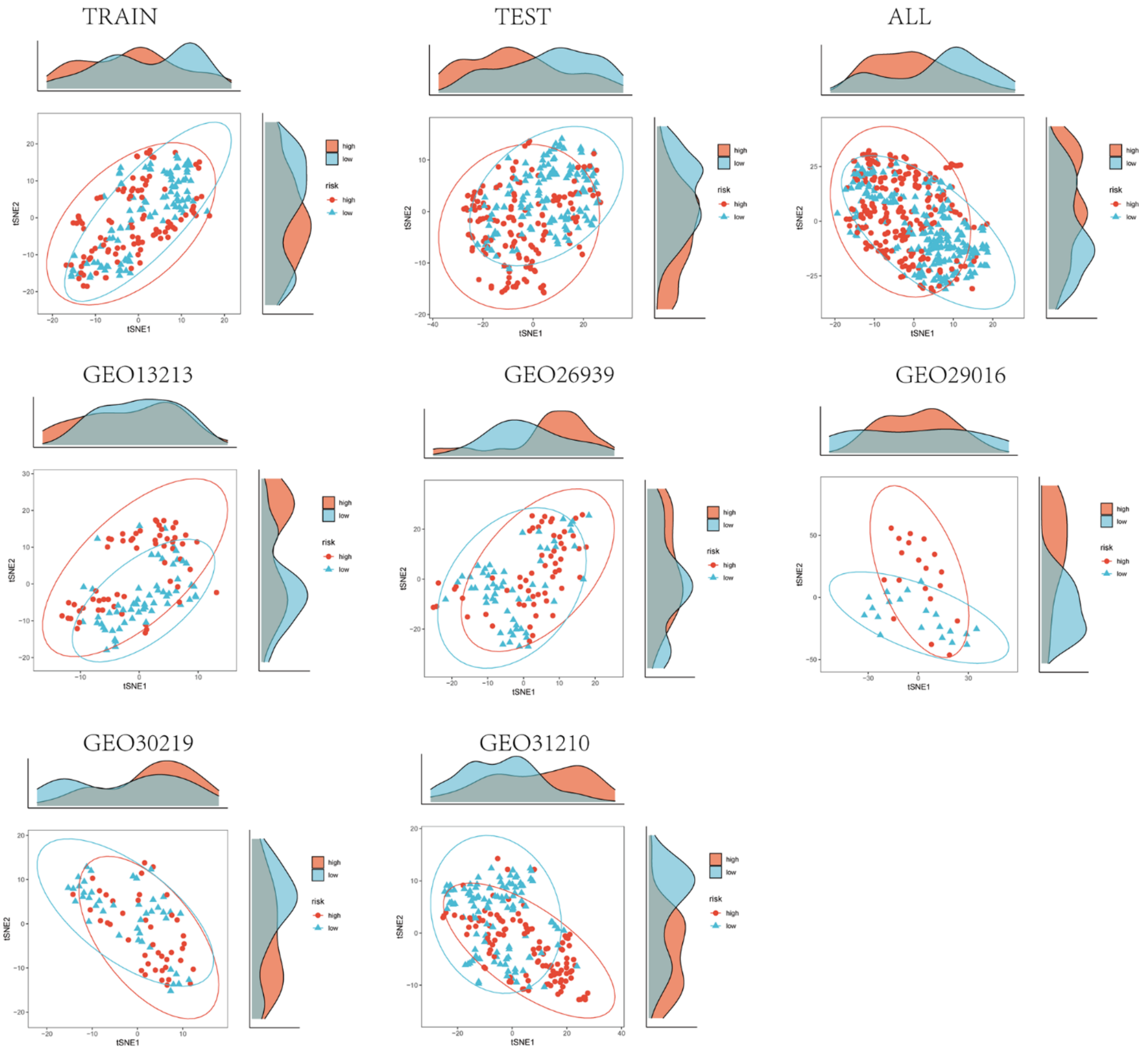

Supplementary Figure 2. t-SNE plot showing the distribution characteristics of samples in bulk RNA-seq.

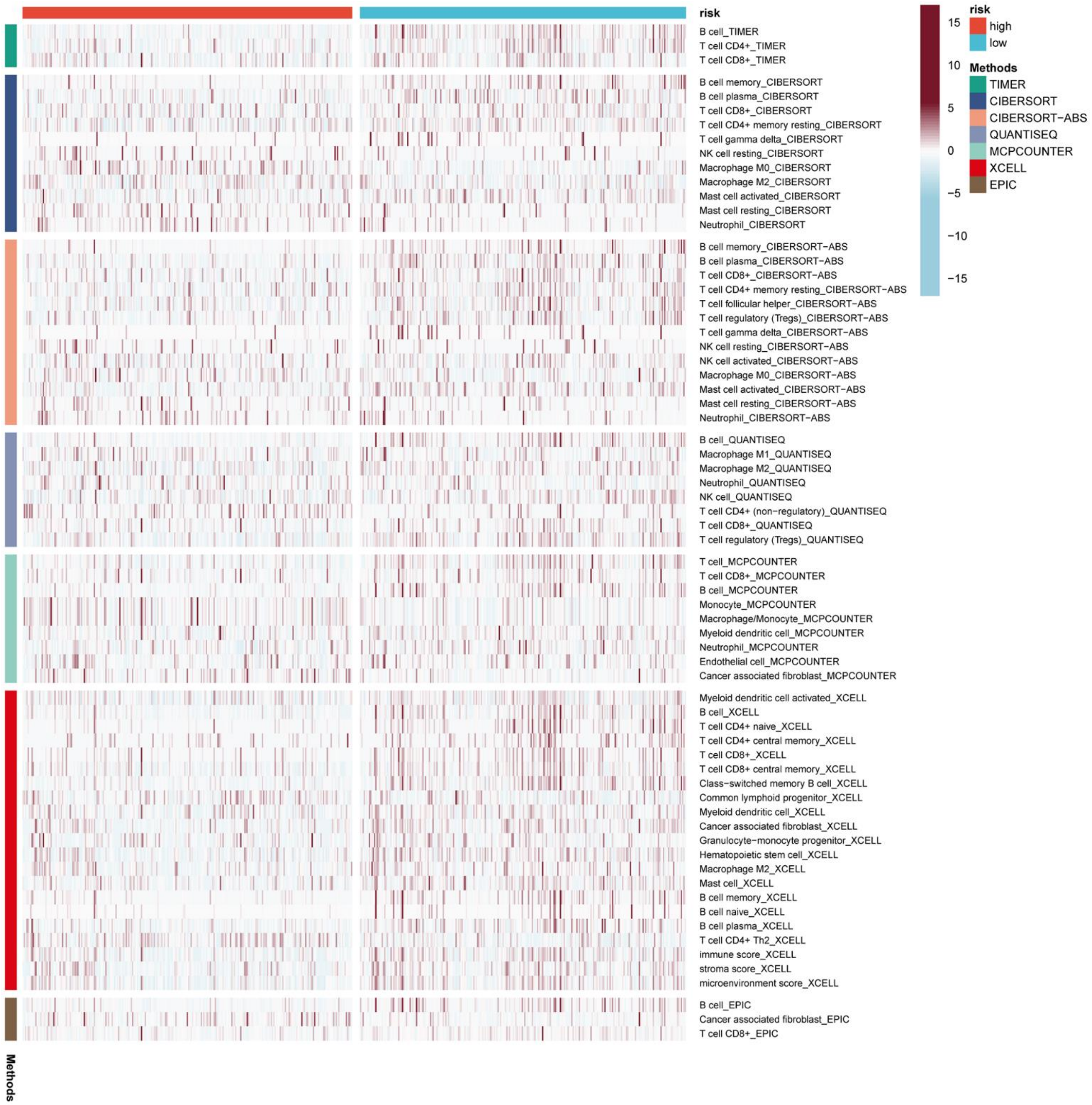

Supplementary Figure 3. Assessing differences in immune cell infiltration in high- and low-risk groups using 7 algorithms.
