# Supplementary material for: The integrated single-cell analysis developed an immunogenic cell death signature to predict lung adenocarcinoma prognosis and immunotherapy
Source: Aging (Albany NY). 2023 Oct 4;15(19):10305–29. doi: 10.18632/aging.205077 (PMC10599752; doi:10.18632/aging.205077)
Supplement: Supplementary Table 1 [file aging-15-205077-s002.pdf]

SUPPLEMENTARY TABLE

Supplementary Table 1. Genes involved in immunogenic cell death.

ATG5
BAX
CALR
CASP1
CASP8
CD4
CD8A
CD8B
CXCR3
EIF2AK3
ENTPD1
FOXP3
HMGB1
HSP90AA1
IFNA1
IFNB1
IFNG
IFNGR1
IL10
IL17A
IL17RA
IL1B
IL1R1
IL6
LY96
MYD88
NLRP3
NT5E
P2RX7
PDIA3
PIK3CA
PRF1
TLR4
TNF
